# Visceral pleural biopsy under medical thoracoscopy for the diagnosis of lung adenocarcinoma

**DOI:** 10.1002/rcr2.616

**Published:** 2020-07-02

**Authors:** Satoshi Terashita, Keiichiro Suminaga, Hiroaki Kawachi, Susumu Noguchi, Tatsuyoshi Ikeue, Takakazu Sugita

**Affiliations:** ^1^ Department of Respiratory Medicine Japan Red Cross Wakayama Medical Center Wakayama Japan

**Keywords:** Biopsy, lung adenocarcinoma, medical thoracoscopy, thoracoscopy, visceral pleura

## Abstract

Medical thoracoscopy is a minimally invasive single‐port endoscopic technique that provides direct visualization of the pleural surface and allows for diagnostic procedures. The diagnostic yield of medical thoracoscopy is high and is generally based on parietal pleural biopsy findings. Pleural biopsies are valuable for a diagnosis. However, visceral pleural biopsies are uncommon because of the risk of prolonged air leak. In this study, we report a rare case of the successful diagnosis of lung adenocarcinoma, based on the findings of visceral pleural biopsy under medical thoracoscopy. To avoid lung injury and pneumothorax, we focused on maintaining the thoracoscope and biopsy forceps in a straight angle as much as possible. While looking straight ahead at the visceral pleural nodule as closely as possible, biopsy samples were carefully obtained while confirming that the normal lung was not held. With careful consideration, visceral pleural biopsies may expand the diagnostic capability of medical thoracoscopy.

## Graphical abstract

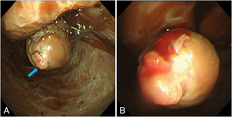

## Introduction

Medical thoracoscopy is increasingly used for diagnosing pleural effusion of unknown aetiology. The British Thoracic Society guidelines recommend thoracoscopy as the next diagnostic test in patients with exudative pleural effusion of unknown aetiology, after an inconclusive thoracentesis [1]. The diagnostic yield of medical thoracoscopy is high and generally results from parietal pleural biopsy findings. Pleural biopsies are valuable for diagnosis. However, visceral pleural biopsies are uncommon. In this study, we report a rare case of a successful diagnosis of lung adenocarcinoma, based on the findings of visceral pleural biopsy under medical thoracoscopy.

## Case Report

An 83‐year‐old woman with a medical history of developmental disorders presented to the emergency outpatient unit with a one‐week history of dyspnoea. Her vital signs were normal and oxygen level (peripheral capillary oxygen saturation, SpO_2_) was 94% on room air. Her physical examination was remarkable for heavily decreased breath sounds in the left lung.

**Figure 1:**
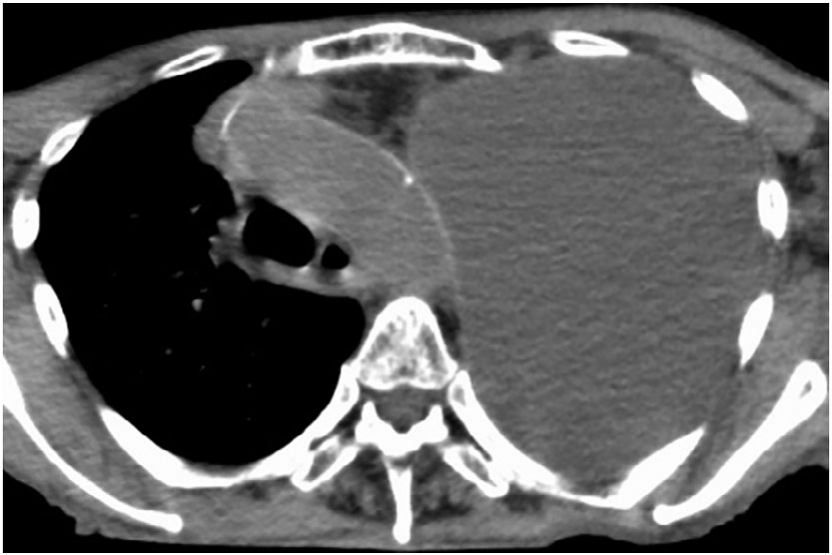
The computed tomography scan of the chest shows large left‐sided pleural effusions.

The patient's chest radiograph and computed tomography scan of the chest showed large pleural effusions on the left lung (Fig. 1). The serum carcinoembryonic antigen level was 84.2 ng/mL. Coagulation tests were within the normal range.

**Figure 2:**
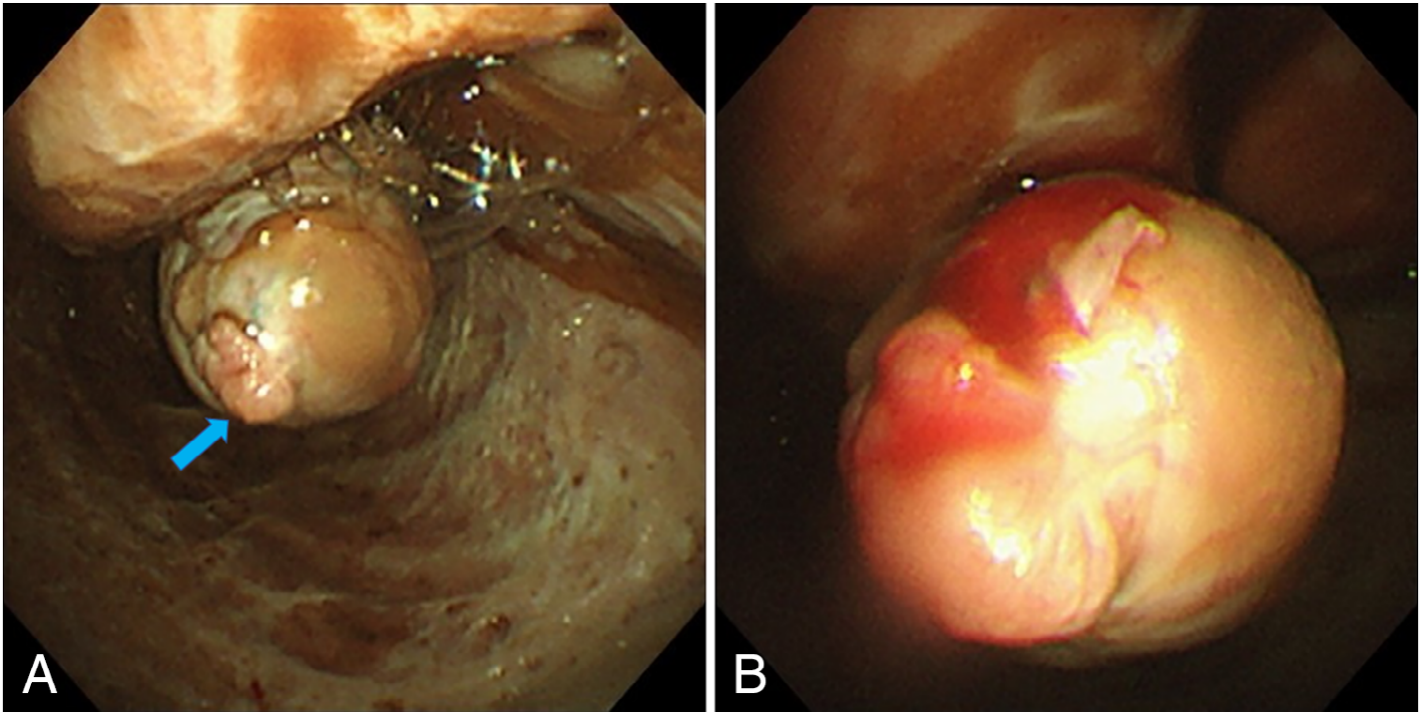
Medical thoracoscopic findings. (A) The arrow indicates a visceral pleural nodule, which is compatible with pleural dissemination. The right bottom of the image shows diffuse thickening of the parietal pleura. (B) After the biopsy, only a small amount of bleeding occurred without any other complications.

Two diagnostic thoracocenteses were non‐diagnostic. Medical semirigid thoracoscopy was therefore performed. Thoracoscopy revealed diffuse thickening of the parietal pleura and the visceral pleura, which had a nodule that was compatible with pleural dissemination (Fig. 2A). We attempted a biopsy of the parietal pleural thickening; however, the procedure was difficult to perform because of flat lesions. Therefore, we attempted a biopsy of the visceral pleura nodule. We carefully observed the surface of its nodule and confirmed an avascular area. To avoid lung injury and pneumothorax, we focused on maintaining the thoracoscope and biopsy forceps in a straight angle as much as possible. While looking straight ahead and as closely as possible at the visceral pleural nodule, biopsy samples were carefully obtained while confirming that the normal lung was not held. After the biopsies, we completed the medical thoracoscopy without any complications such as pneumothorax and lung injury. Only a small amount of bleeding occurred (Fig. 2B).

Haematoxylin and eosin staining of the biopsy specimens revealed a malignant neoplasm. Immunohistochemical staining revealed a positive reaction to thyroid transcription factor 1 and negative reactions to calretinin and D2‐40. We consequently diagnosed lung adenocarcinoma.

## Discussion

Medical thoracoscopy is a minimally invasive single‐port endoscopic technique that provides direct visualization of the pleural surface and allows diagnostic procedures and therapeutic procedures to be administered [2]. It has a diagnostic yield of 86% and complication rate of 10%, compared to those of closed pleural biopsy (62% and 17%, respectively) [3]. In a systematic review [4] evaluating the diagnostic accuracy of medical thoracoscopy for patients with pleural effusion of undetermined aetiology, medical semirigid thoracoscopy had a pooled sensitivity and specificity of 97% and 100%, respectively. These values are generally based on parietal pleural biopsy findings. Pleural biopsies are valuable for diagnosis. However, visceral pleural biopsies are uncommon. The British Thoracic Society guidelines indicate that a medical thoracoscopist practicing at level I competence (i.e. most district general physicians) should be able to perform a biopsy of the parietal pleura but not the visceral pleura. Visceral pleural biopsy is listed as a level II technique that should be performed by more experienced practitioners within a unit with a major interest in pleural disease [1]. Researchers in another study [5] assert that pleural biopsy samples should only be taken from the parietal pleura to avoid the risk of prolonged air leak. However, to the best of our knowledge, a successful diagnosis based on the findings of visceral pleural biopsy under medical thoracoscopy has not been reported. In our patient, visceral pleural biopsies under medical thoracoscopy contributed to the diagnosis of lung adenocarcinoma. For the diagnosis, insulation‐tipped diathermic knife for a biopsy of the parietal pleural flat thickening might be worth considering as an alternative.

To avoid lung injury and pneumothorax, we focused on maintaining the thoracoscope and biopsy forceps in a straight angle as much as possible. While looking straight ahead and as closely as possible at the visceral pleural nodule, biopsies were carefully performed while confirming that the normal lung was not held. This confirmation seems to be essential for safety. With careful consideration, visceral pleural biopsy may expand the diagnostic capability of medical thoracoscopy.

### Disclosure Statement

Appropriate written informed consent was obtained for publication of this case report and accompanying images.
